# Composite 3D printing of biomimetic human teeth

**DOI:** 10.1038/s41598-022-11658-y

**Published:** 2022-05-12

**Authors:** A. J. Cresswell-Boyes, G. R. Davis, M. Krishnamoorthy, D. Mills, A. H. Barber

**Affiliations:** 1 Dental Physical Sciences Unit, Centre of Oral Bioengineering, Institute of Dentistry, Barts and the London School of Medicine and Dentistry, Queen Mary University of London, Mile End Road, London, E1 4NS UK; 2 Oral Health Innovation, GlaxoSmithKline Consumer Healthcare, St George’s Avenue, Weybridge, KT13 0DE UK; 3 School of Engineering, London South Bank University, Borough Road, London, SE1 0AA UK

**Keywords:** Dentistry, Biomedical engineering, Mechanical engineering, Biomaterials, Structural materials

## Abstract

Human teeth are mechanically robust through a complex structural composite organisation of materials and morphology. Efforts to replicate mechanical function in artificial teeth (typodont teeth), such as in dental training applications, attempt to replicate the structure and morphology of real teeth but lack tactile similarities during mechanical cutting of the teeth. In this study, biomimetic typodont teeth, with morphology derived from X-ray microtomography scans of extracted teeth, were 3D printed using an approach to develop novel composites. These composites with a range of glass, hydroxyapatite and porcelain reinforcements within a methacrylate-based photopolymer resin were compared to six commercial artificial typodont teeth. Mechanical performance of the extracted human teeth and 3D printed typodont teeth were evaluated using a haptic approach of measuring applied cutting forces. Results indicate 3D printed typodont teeth replicating enamel and dentine can be mechanically comparable to extracted human teeth despite the material compositions differing from the materials found in human teeth. A multiple parameter variable of material elastic modulus and hardness is shown to describe the haptic response when cutting through both human and biomimetic, highlighting a critical dependence between the ratio of material mechanical properties and not absolute material properties in determining tooth mechanical performance under the action of cutting forces.

## Introduction

Teeth have outstanding mechanical properties that are yet to be replicated using engineering routes. Current artificial teeth (typodont teeth) are typically made from engineering materials that result in significant mechanical differences from human teeth^1^, although replications of shape and colour have been successfully achieved^2,3^. In dental education biomimetic typodont teeth reduce the use of extracted human teeth for training^3–6^, however, the need to mimic the mechanical properties of human teeth, not just morphology, is significant in most areas of dentistry. Approaches to replicate the mechanical function of human teeth, and more generally of all teeth, is challenging due to the complex dependency between tooth morphology, material composition and material organisation. Attempts to engineer a tooth with comparable mechanical properties must consider all these parameters as well as reconcile manufacturing routes that will be more rapid than the relatively slow biological processes that were employed to develop human teeth. Finally, the mechanical functions are additionally complex with a range of loading regimes experienced by a human tooth^7^. Here, the resistance of a human tooth to mechanical cutting is considered due to its importance in tactile feedback during filling procedures in dental training; a common high-volume usage for typodont teeth. The development of a biomimetic tooth, therefore, requires an understanding of the complex interaction between materials in established manufacturing processes to deliver appropriate mechanical performance comparable to a human tooth.

The range of developmental dental materials is vast, but poly (methyl methacrylate) (PMMA) resin remains the leading candidate in dentistry due to its high availability, low cost, biocompatibility and acceptable aesthetics^8^. PMMA material is relatively high in performance^9^, processable in a range of manufacturing processes^10^, biocompatible^8^ and, as a result, frequently used in denture production^11^. However, PMMA has been observed to fail easily due to fatigue or excessive mastication, making PMMA dentures hard to repair^12^. The addition of fillers within PMMA to produce a composite has been noted as improving the impact strength of dentures but preparation is difficult as the filler increases the viscosity of the PMMA, thus reducing its ability to flow into the desired shape^12,13^. Producing composites with relatively hard and soft materials is therefore advantageous in mimicking the varying material composition of hydroxyapatite within the hard enamel and softer dentine regions of the human tooth^7^. Despite the vast literature on improving dentures, creating biomimetic typodont teeth with mechanical properties that provide realistic tactile responses in dental education applications, such as the “feel” when cutting teeth, is minimal. Reymus, Stawarczyl^14^ investigated the suitability of several 3D printed typodont teeth compared with commercial typodont teeth by evaluating their hardness values and dentists’ opinions on mechanical instrumentation (“feel”) of the dentine. The authors concluded that the tooth replicas were unable to recreate human dentine. Despite the “feel” of typodont teeth being evaluated qualitatively, there was no quantitative analysis carried out. It is worth noting, that previous studies have not carried out this quantitative analysis.

The development of biomimetic typodont teeth that mimic the mechanical properties of the native tooth requires investigations into controllable materials processing and composition that define resultant tooth performance. Therefore, multiple studies have investigated the addition of different fillers in manufacturing PMMA-based artificial teeth^8,10–12^. Applying increasing filler content to produce a composite has improved the mechanical properties such as hardness and fracture resistance^12^. However, the ineffectiveness of a biomimetic tooth is often captured in literature, particularly where a higher tactile perception threshold (a perceived feeling that it is more difficult to cut typodont teeth compared to natural)^15–17^ is found for typodont teeth in clinical training when compared to native extracted teeth^15^.

Many studies look to produce mimetic materials by recreating the hardness of natural teeth but, critically, no studies exist that stress the importance of relative material properties over absolute properties in defining overall mechanical performance. For contacts between a typodont tooth and cutting device, Amini and Miserez^18^ provided a key relationship between hardness (*H*) and elastic modulus (*E*) in explaining the wear and abrasion properties of materials, with the ratio of $\frac{{H}^{3}}{{E}^{2}}$ defining the magnitude of resistance to fracture between two contacting bodies. Therefore, a typodont tooth is able to mimic human teeth by control of the relative hardness and elastic modulus of the component materials instead of replicating the absolute hardness and elastic modulus of native tissue.

Advances in 3D printing offer a manufacturing route to create typodont teeth that are morphologically accurate and, through composite material development, provide a range of mechanical properties. Senatov, Niaza^19^ and Corcione, Gervaso^20^ both developed hydroxyapatite (HAp) – polylactic acid composite materials for 3D printing for use in tissue engineering and creating porous scaffold suitable for bone grafts. Both studies found an increase in mechanical properties via compressive strength without a negative effect on the rheological performance. Despite the improvements in mechanical properties, the authors were unable to match that of the native tissue. The use of high-resolution 3D imaging techniques in conjunction with 3D printing is an established workflow that allows for an accurate recreation of the geometry of the original object. For example, Reymus, Fotiadou^3^ produced an available workflow for dental educational institutions with access to CBCT and 3D printing facilities to create resin teeth for endodontic teaching purposes. Combining these techniques with material development is expected to create an accurate representation of natural teeth. However, a common drawback of these imaging techniques is the inclusion of image noise and ring artefacts or beam hardening artefacts which may interfere with the image acquisition, requiring more input at the image post-processing stage^21,22^. Therefore, the aim of this study is to 3D print typodont teeth with materials developed to mimic both the morphology and mechanical response of natural teeth. X-ray microtomography (XMT) is used here to image natural teeth at high resolution to accurately map the geometry of the samples. XMT was chosen as the imaging technique due to its non-destructive nature and the ability to easily segment data between structures, as Parwani, Curto^23^ established when segmenting bone as well as 3D printed replicas. The imaging data can be manipulated and converted to a suitable format for 3D printing^24^. A force measuring system was further developed for this study to enable mechanical evaluation of cutting teeth as a method to assess the success of the 3D printed biomimetic typodont teeth compared to their extracted equivalents. This study will therefore justify a novel haptic technique used to measure the forces imposed during cutting on extracted teeth and typodont teeth from a dental handpiece.

## Results

**Figure 1:**
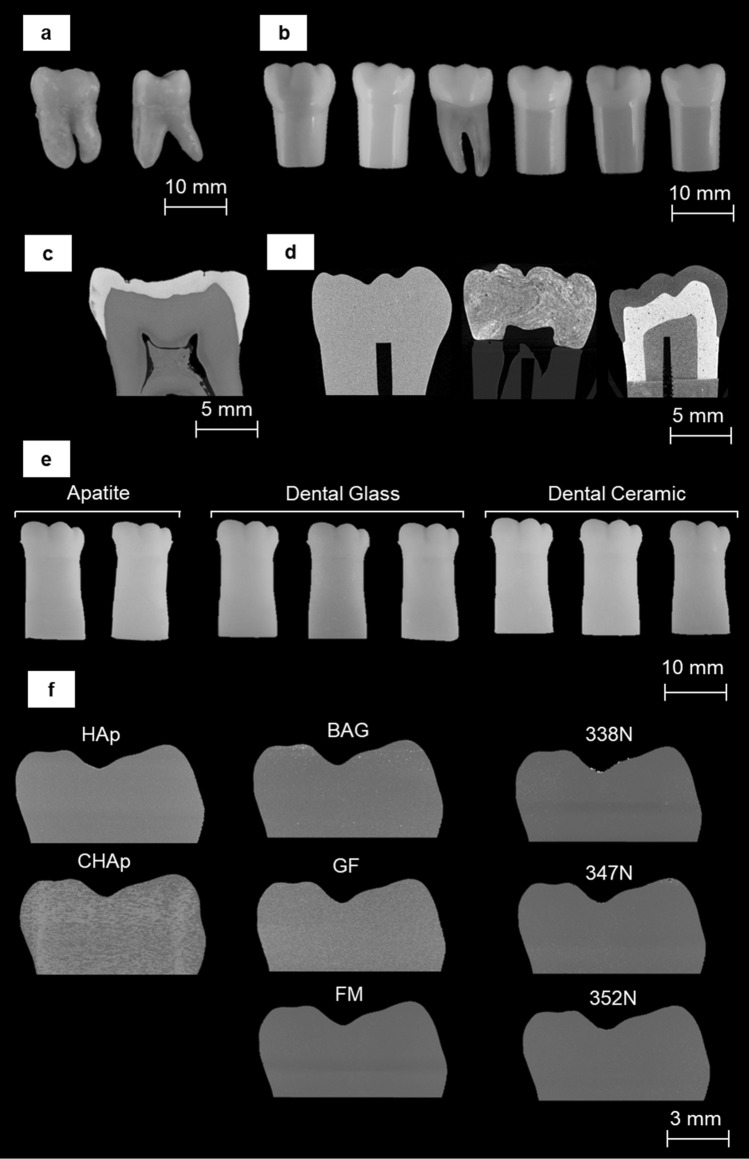
Reconstructed XMT images of extracted, commercially available typodont teeth and 3D printed typodont teeth, highlighting the differences in material, distribution of particle fillers and structures. (**a**) Extracted mandibular molar. (**b**) Commercially available typodont teeth. (**c**) Reconstructed XMT images of the extracted mandibular molars. (**d**) Reconstructed XMT image of Acadental, Fabrica de Sorrisos, and Nissin (*left to right*). (**e**) Three material groups of 3D printed composites; apatites, dental glass and dental ceramics. (**f**) Reconstructed XMT images of the 3D printed composites; hydroxyapatite (HAp), carbonated hydroxyapatite (CHAp), bioactive glass (BAG), glass flake (GF), fluormica glass (FM), and dental ceramic (338 N, 347 N, 352 N).

XMT is adept at fully describing the morphology of teeth and produced high-contrast images highlighting the differences in structures between extracted and typodont teeth (Fig. 1c-d). The high-contrast scan clearly shows the difference between the highly mineralised enamel shown as the bright region in the image and the less mineralised dentine. One of the commercial typodont teeth (Frasaco) shows a single contrast, suggesting the use of one material for both enamel and dentine, or two materials with the same X-ray opacity. The remaining typodont teeth all differentiated between enamel and dentine by using different materials that result in differing contrast in the XMT images in Fig. 1d.

Figure 1f shows reconstructed XMT images of the 3D printed materials. All images demonstrate a uniformity in geometry and size, with a varying grey-level due to the additional filler particles. The reconstructed XMT images show the distribution of filler particles (at 25 wt. %) within the 3D printed constructs but highlighting a consensus for the particles to remain distributed within the layers, as particularly seen in the carbonated hydroxyapatite (CHAp) image.

**Figure 2:**
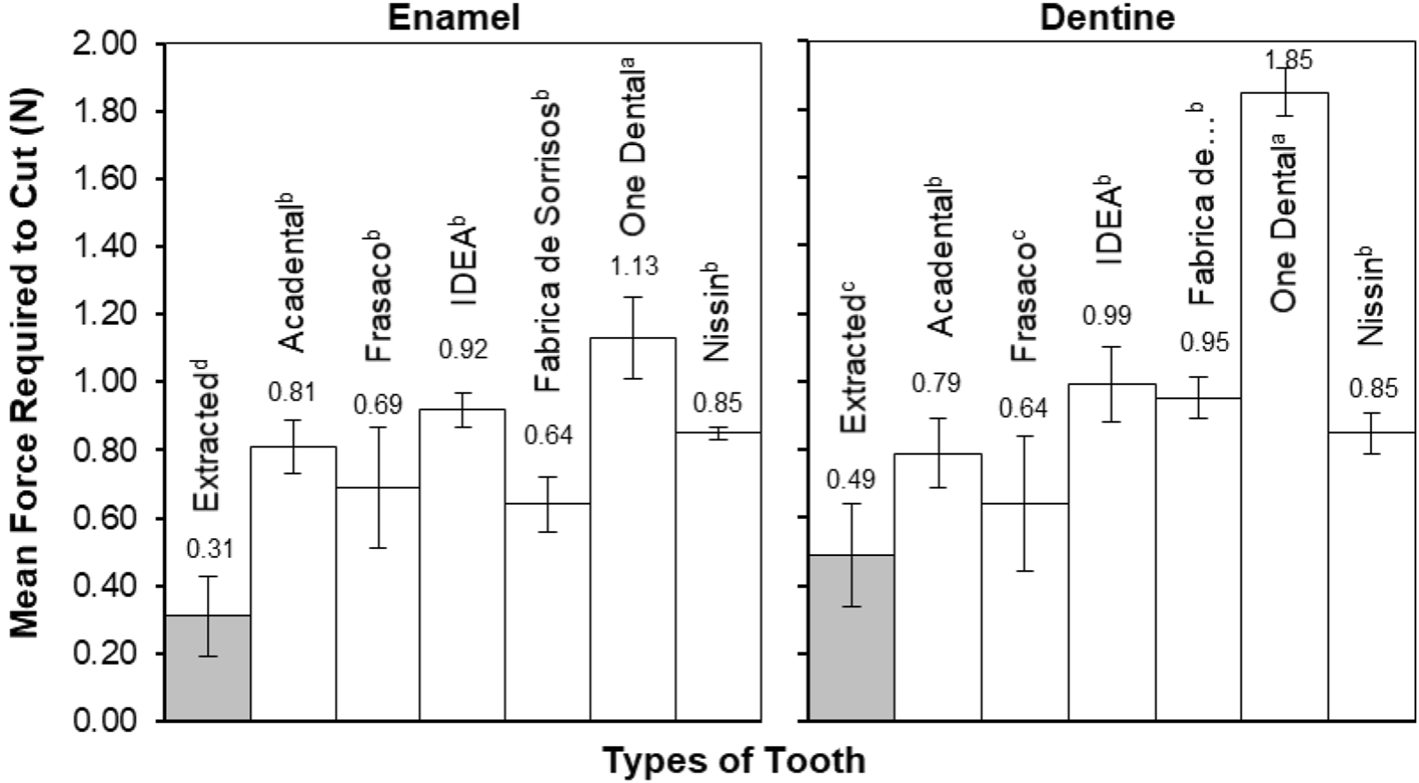
The mean force required to cut extracted and typodont teeth, highlighting the forces required for both enamel and dentine materials. Error bars are presented as the standard deviation of the data. *n* = 8. Subscript denotes statistical differences between groups, a, b, c.

Figure 2 presents the mean force required to cut the extracted and commercially available typodont teeth. Extracted enamel required the least amount of force to cut, with 0.31 N (± 0.12) required compared to the range from typodont teeth 0.69 N (± 0.18) (Frasaco) to 1.13 (± 0.12) (One Dental). The same trend in forces was seen also in the dentine regions across the various samples, with extracted dentine requiring 0.49 N (± 0.15), whereas 0.64 N (± 0.20) (Frasaco) to 1.85 N (± 0.07) (One Dental) was required to cut the commercial typodont teeth. Statistical analysis carried out in the form of one-way ANOVA indicated a significant difference (*P* < 0.05) between extracted samples and the artificial products, which was true for both enamel and dentine. No significant difference was noted between the commercial typodont teeth, with the exception of One Dental, which exhibited double the force of other commercial typodont teeth, and more than four times that of extracted tissue. Significantly larger cutting forces were consistently observed for the One Dental samples compared to the other commercially available groups.

**Figure 3:**
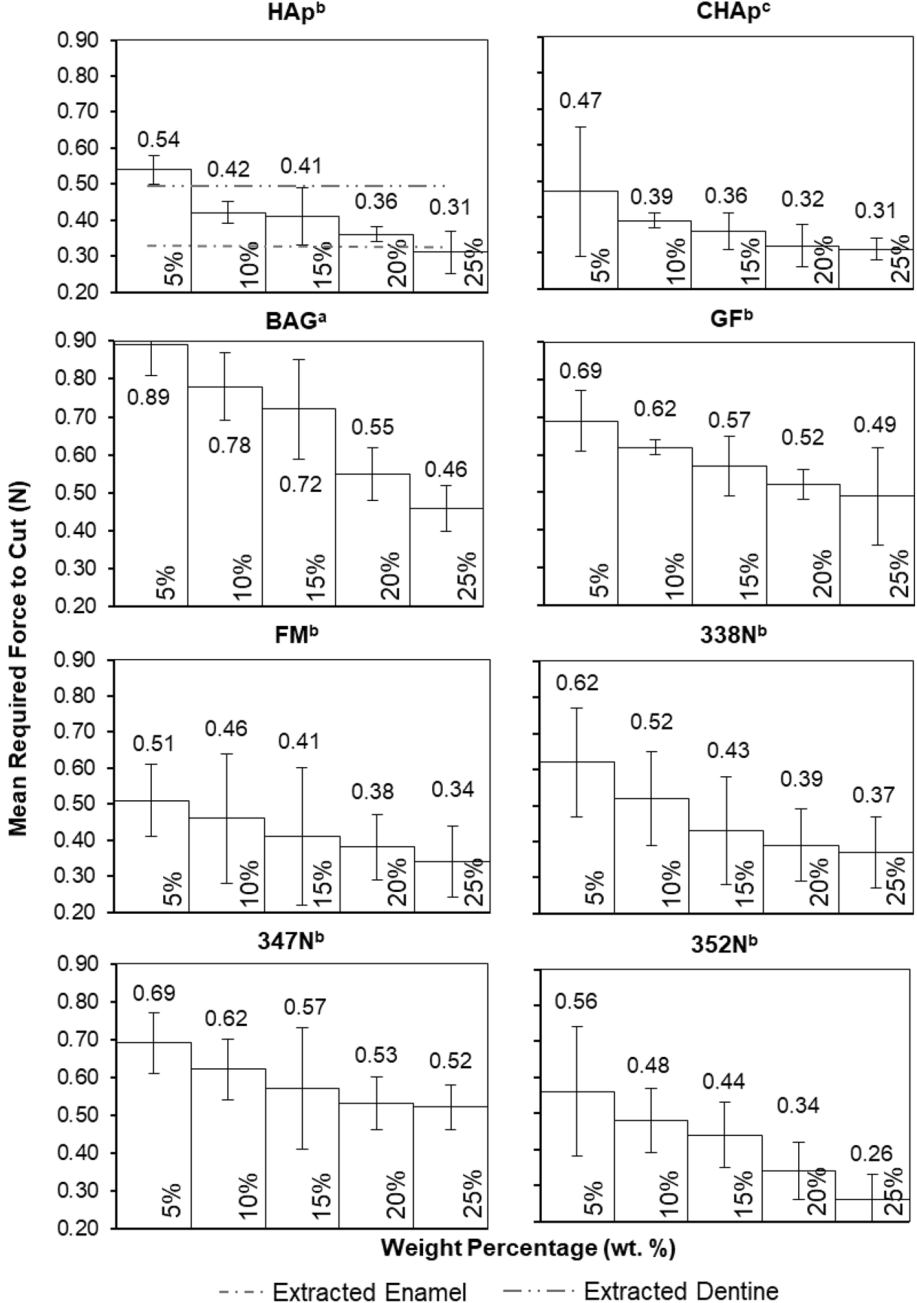
The mean force required to cut 3D printed teeth, highlighting the forces required for all materials. Error bars are presented as the standard deviation of the individual samples. *n* = 8. Subscript denotes statistical differences between groups, a, b, c.

Figure 3 shows the required forces to cut the developed 3D printed materials, presenting the range of materials evaluated in this study as well as the different weight percentages of reinforcement used to create the 3D printed typodont teeth. A decrease in cutting force as the weight percentage of reinforcement is increased is observed across all samples. Of the 40 different compositions, three compositions closely matched (± 0.02 N) the forces required to cut extracted enamel (0.31 N), specifically 25 wt. % HAp (0.31 N ± 0.06), 20 wt. % CHAp (0.32 N ± 0.06) and 25 wt. % CHAp (0.31 N ± 0.03). Four compositions closely matched (± 0.02) the forces required to cut (0.49 N ± 0.15) extracted dentine; 25 wt. % glass flake (GF) (0.49 N ± 0.13), 5 wt. % CHAp (0.47 N ± 0.18), 5 wt. % fluormica glass (FM) (0.51 N ± 0.10), and 10 wt. % Vitadur 352 N (0.48 N ± 0.09). Statistical analysis showed a significant difference (*P* < 0.05) between 5 wt. % and 25 wt. % reinforcement samples for all material groups, demonstrating a difference when altering, not only the reinforcement material but also the wt. %. Between groups, bioactive glass (BAG) and CHAp overall, were statistically different to the other composites, with BAG and CHAp also being statistically different from each other. BAG, overall, giving the highest forces required to cut, with CHAp, overall, giving the lowest.

## Discussion

The force experiments carried out established that more force was required to cut commercial typodont teeth compared to extracted (Fig. 2). 0.31 N was needed to cut extracted enamel whereas more than double the force was required to cut the lowest force of artificial enamel, 0.64 N (Fabrica de Sorrisos). Moreover, the force more than tripled when compared to the highest force of 1.13 N (One Dental) recorded for typodont teeth. This discrepancy in forces may explain why undergraduate students dislike the use of typodont teeth^14,15^, as such typodont teeth do not have the same tactile response as extracted teeth. However, this discrepancy does not explain why more force was required to cut typodont teeth. The discrepancy between the cutting forces applied to artificial and extracted teeth was initially hypothesised as due to residual plastic material adhering to the bur during cutting, possibly from localised heating that could cause an increase in the bur-plastic contact area and a corresponding increase in cutting force. An inspection of the burs after cutting showed little evidence of material remaining on the bur after cutting, probably due to the use of irrigation throughout the cutting procedure. Therefore, the additional force required to cut typodont teeth is expected to be due to the mechanical performance of the materials under the cutting action rather than the clogging of the bur.

An observed decrease in force required to cut each sample with increased wt. % is found in Fig. 3 and requires further explanation. As more particulates are added, the amount of polymer is decreased, simply making the material more brittle due to the composition of the composite becoming dominated more by the increasing volume fraction of particulate. In terms of mechanical properties, the addition of more particulates increases hardness substantially and elastic modulus slightly. Furthermore, due to the nature of the DLP manufacturing process, objects are produced via layers as observed from the XMT images (Fig. 1). The presence of these layers, open the objects up to delamination, which would require less force to cut compared to the commercial typodont teeth, which are injection moulded, producing objects as solid entities, as shown in Fig. 1b.

**Figure 4:**
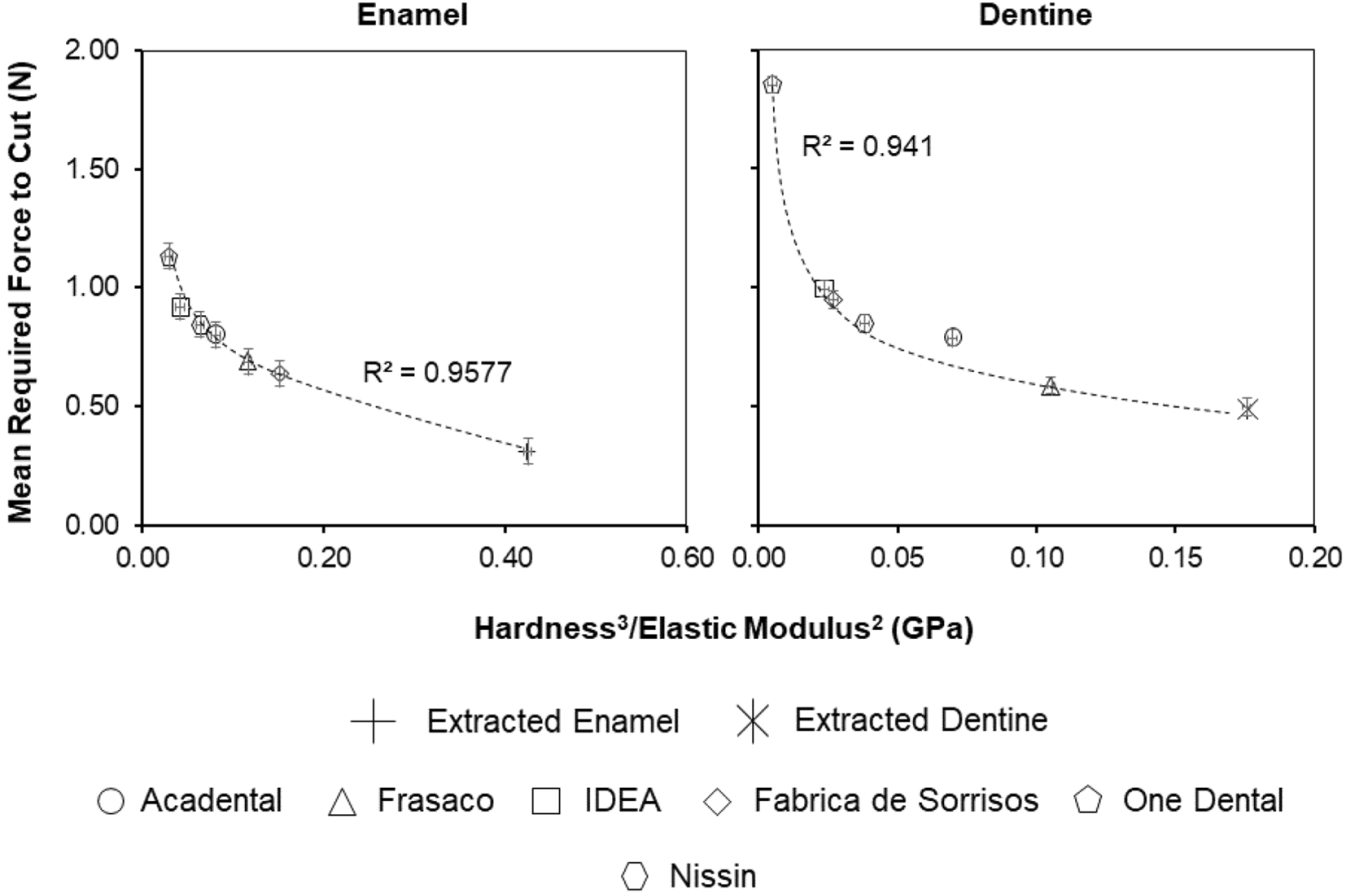
Average force required to cut extracted and typodont teeth against $\frac{{H}^{3}}{{E}^{2}}$. Trendline and R^2^ values refer to typodont teeth, excluding the extracted teeth values. Error bars are presented as the standard deviation of the data. n = 8.

As previously mentioned, work by Amini and Miserez^18^ investigated the wear and abrasion resistance of biological materials based on their mechanical properties and, specifically, the ratio of hardness (*H*) to elastic modulus (*E*) defined as $\frac{{H^{3} }}{{\overline{E}^{2} }}$. The authors established this ratio property to describe the increased material energy to fracture as a (blunt) contact area decreased. This ratio was established in Miserez, Li^25^ to understand how unmineralised squid beak could break down mineralised objects such as shells and bone. Therefore, using the ratio established by Miserez, Li^25^, the hardness and elastic modulus values were plotted against the average force required to cut as shown in Fig. 4. Hardness and elastic modulus values of base materials were collected using Vicker’s microhardness indentation and compression testing, respectively. The plot indicates a correlation between the cutting force and $\frac{{H}^{3}}{{E}^{2}}$, proposed above, that governs failure of a material under a mechanical contact. A negative correlation is seen between force and the properties ratio in Fig. 4, as the average cutting force decreases as the material properties ratio increases. This relationship can be summarised as the less compliant material requiring less force to cut or fracture the dental materials. Such an observation was also seen in Miserez, Li^25^ and Amini and Miserez^18^, as materials with a higher $\frac{{H}^{3}}{{E}^{2}}$ ratio tended to be brittle with enamel having a ratio of 7.0–9.0 MPa and dentine 0.3–1.3 MPa. These ratio values in literature are low compared to the ratios seen in this study; 0.42 and 0.18 GPa for enamel and dentine, respectively, as shown in Fig. 4. However, the studies here consider a more complex mechanical loading condition due to the contact with materials occurring using a rotating tool, suggesting that direct comparison is difficult, but the general contact failure condition defined by the properties’ ratio is still relevant. Critically, the ratio of mechanical properties is observed as the key to determining the cutting force and not the absolute properties of the materials. The use of 3D printed materials with mechanical properties that deviate substantially from enamel and dentine can, therefore, provide the same haptic response of material removal through selection of appropriate $\frac{{H}^{3}}{{E}^{2}}$ values.

**Figure 5:**
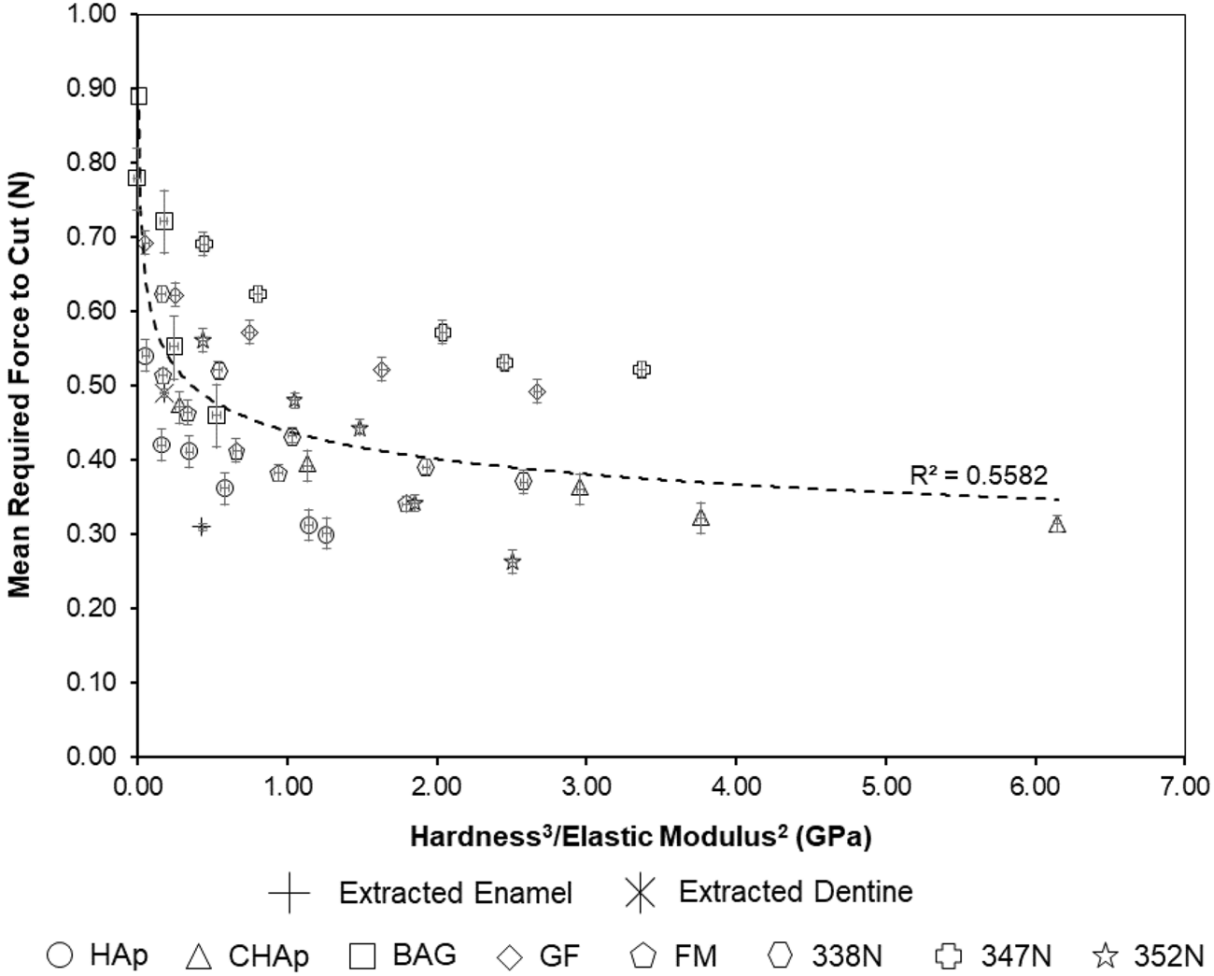
Average force required to cut 3D printed typodont teeth against $\frac{{H}^{3}}{{E}^{2}}$. Trendline and R^2^ values refer to typodont teeth, excluding the extracted teeth values. Error bars are presented as the standard deviation of the data. n = 8.

Figure 5 shows the mean force required to cut the 3D printed typodont teeth against the $\frac{{H}^{3}}{{E}^{2}}$ ratio. A trend was observed for the 3D printed typodont teeth prepared in this study where increasing the wt. % resulted in a decrease in the cutting force, which was consistent across the range of material groups. Variations in data from the trend line are possibly due to the presence of layers within the printed composites, which are not considered in our assumptions. Vitadur 352 N composites showed the biggest percentage decrease in force (53.57%) as wt. % increased, with GF composites showing the least change, 28.99%. Similar forces (± 0.02 N) seen between the 25 wt. % HAp, 20 wt. % CHAp, 25 wt. % CHAp, and extracted enamel. For extracted dentine, 25 wt. % GF, 5 wt. % CHAp, 5 wt. % FM, and 10 wt. % Vitadur 352 N had similar forces (± 0.02 N), as well as similar ratios. Our results, therefore, indicate that HAp, CHAp and GF, CHAp, FM, 352 N composites require cutting forces that are analogous to those required to cut through natural human enamel and dentine, respectively.

In conclusion, typodont teeth used for dental training reportedly require more force to cut than natural teeth. Quantitative evaluation confirmed that the applied cutting force could be up to four times as high. 3D-printable materials can be tailored such that the required cutting force more closely matches that for enamel and dentine. With geometry obtained from X-ray microtomography of real teeth, typodont teeth have been made which give better visual and haptic cues appropriate for training dentists.

## Methods

### Specimen selection

A variety of tooth samples were examined in this study and were grouped into three;

1. Extracted teeth, as a baseline to test how typodont teeth compare to these natural teeth.
2. Commercially available typodont teeth.
3. Typodont teeth developed in this work using a 3D printing of composite materials approach.

Multiple non-carious extracted mandibular first molars were selected for the study to recreate through 3D printing. Extracted teeth were selected from a human tissue bank; with ethical approval obtained from Queen Mary Research Ethics Committee (QMREC2008/57). Six commercial artificial mandibular first molars were selected from different suppliers that are commonly used within dental schools in the UK; Acadental (USA), Frasaco (Germany), IDEA (USA), Fabrica de Sorrisos (Brazil), One Dental (Australia) and Nissin (Japan). Composite tooth development is described below.

### X-ray microtomography

Specimens were imaged using the MuCAT2 time-delay integration (TDI) high-contrast resolution scanner developed by Davis and Elliott^26^. MuCAT2 utilises a charge-coupled detector camera (Spectral Instruments, USA) with a 100 μm thick columnar caesium iodide scintillator (Applied Scintillation Technologies Ltd., UK)^24,26,27^. Natural teeth were scanned at 90 keV, 180 μA, whilst typodont teeth, being less X-ray opaque, were scanned at 40 keV, 405 μA (Fig. 1). The composite materials developed in the study were later imaged using the same settings as the commercial typodont teeth. All specimens were imaged at a 15 μm voxel size. Images were reconstructed using a modified Feldkamp cone-beam back-projection algorithm^28^. The reconstructed images were then modified and converted into a 3D printing file format (*.stl) as described in a previous study^24^. A physical map of the tooth used for 3D printing typodont teeth, can be found in the supplementary material in the form of a *.stl file.

### Composite development

Commonly used materials in a dental clinical and research setting were selected to create a composite material. Such materials included HAp, CHAp, BAG, GF, FM and ceramic materials used in the manufacturing of dentures of different hardness. Details of materials production are outlined in Table 1.

**Table 1:** List of base powder materials used to fabricate typodont teeth.

Material	Manufacturer
Capital® Sintered Hydroxyapatite	(Plasma Biotal Ltd., UK)
Carbonated Hydroxyapatite	Produced as outlined in Landi, Gelotti^29^
45S5 Bioglass®	Produced as outlined in Lefebvre, Geremillard^30^
Glass Flake	(Glass Flake Ltd., UK)
Fluormica Glass	Produced as outlined in Rashwan, Cattell^31^
Vitadur® Alpha 337 N Porcelain	(Vita Zahnfabrik, H. Rauter GmbH & Co., Germany)
Vitadur® Alpha 348 N Porcelain	(Vita Zahnfabrik, H. Rauter GmbH & Co., Germany)
Vitadur® Alpha 352 N Porcelain	(Vita Zahnfabrik, H. Rauter GmbH & Co., Germany)

All powders were ground using a Gy-Ro mill (Glen Creston, UK) for 10 min, before being sieved through a < 38 μm stainless steel sieve (Endocotts Ltd., UK). The powders were then added to a photopolymerised polymer resin (Anycubic 405 nm Rapid Resin, Anycubic, China). The resin itself is a mixture of acrylate oligomer, acrylate monomer, methacrylate monomer and a photoinitiator. The powder was added to the resin at different weight percentages (5, 10, 15, 20 and 25) and the “slurry” was then mechanically mixed for 24 h at 37 °C to allow for complete dispersion. The mixture was placed within an opaque container to ensure no curing took place before printing.

### Digital light processing

A digital light processing (DLP) printer was used (Anycubic Photon, Anycubic, China) to produce the composites. Due to the experimental nature of the materials manufactured a low-cost printer was chosen. It is worth noting that the use of these experimental materials caused no apparent detrimental effect to the printer such as damage to the vat film layer or the build platform. Each composite was printed at a layer height of 50 μm, with each layer cured for 25 s. These settings were set using the printer’s slicing software Anycubic Photon Slicer (Version 1.3.3, 2017; Anycubic, China). Overall, printing 20 individual mandibular molars took 4 h. Printability of tooth geometry was tested by producing molar shapes rather than cylindrical samples, as well as to ensure comparison with force measurements on native tissue. Once printed, the models were washed in 90% ethanol for 20 min to remove any uncured resin, then further cured for 30 min at 60 °C using a Formlabs Wash and Formlabs Cures respectively (Formlabs Inc., USA).

Once potential enamel and dentine analogues were identified through the force measurements, separate enamel and dentine structures were printed using the identified materials. The enamel was printed oversized (by 2%), to allow the fitting of the dentine inside. The separate structures were fitted together using uncured “enamel” resin placed on the inside of the enamel, the dentine was then fixed to the enamel by a further curing for 10 s using a handheld UV cure device (3 M™ Elipar™ DeepCure-L LED Curing Light, The 3 M Company, USA).

### Force measurements

**Figure 6:**
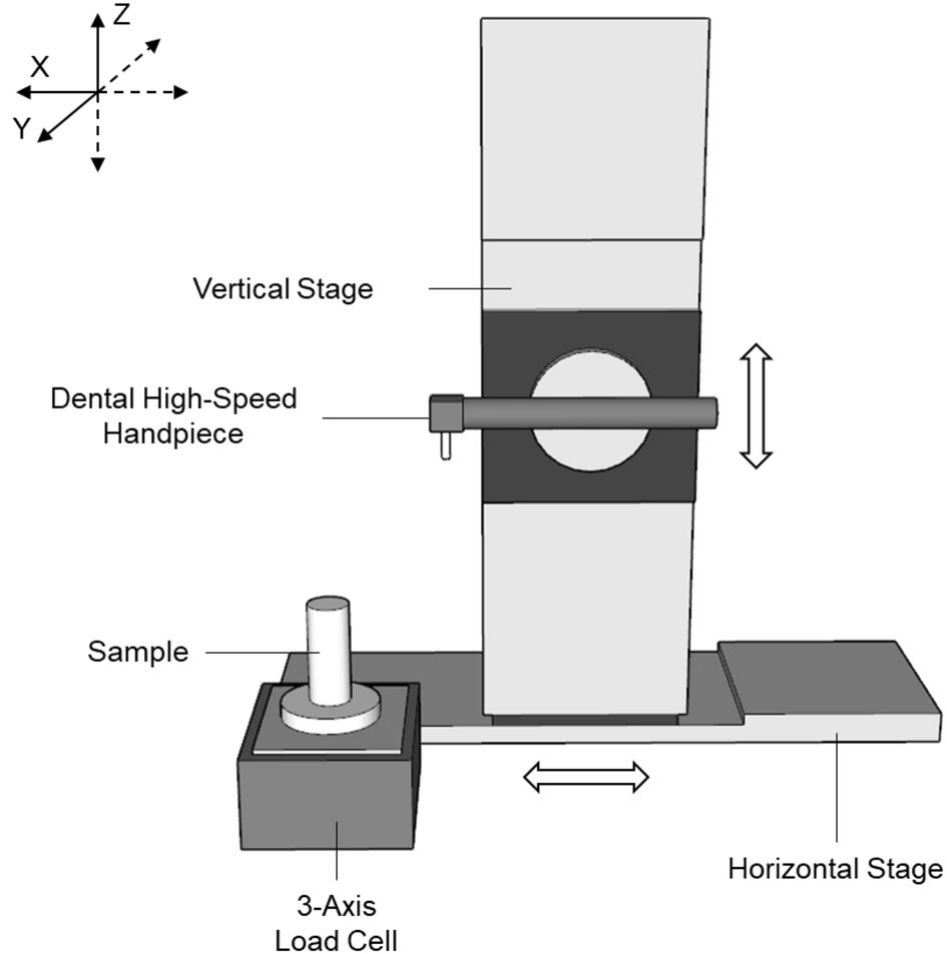
Schematic diagram and picture of the automatic force stage set-up. Directions were measured in three, *X* = Mesiodistal, *Y* = Buccolingual and *Z* = Occlusal.

**Figure 7:**
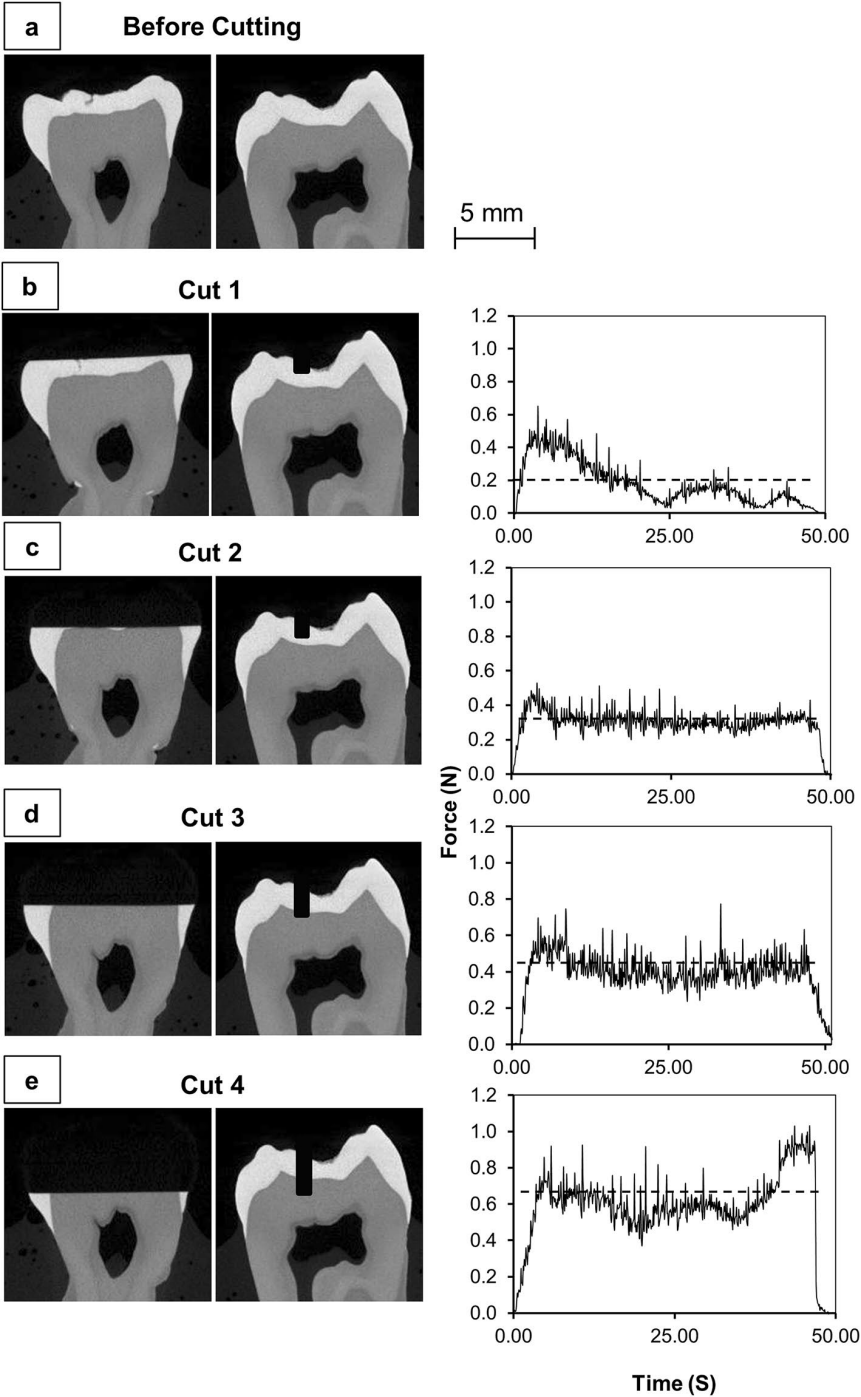
An extracted mandibular molar was imaged at 30 µm resolution using XMT before and after cuts, showing the amount of material removed from each 1 mm deep cut. (**a**) Before cutting. (**b**) After cut 1. (**c**) After cut 2. (**d**) After cut 3. (**e**) After cut 4. The force data shows the difference in force required to cut between cuts, with the dotted line in the graphs showing the mean force required. All graphs are set to the same scale.

The ability to establish a measurement for the tactile perception experienced by training and qualified dentists does not appear to have been investigated previously. Therefore, a novel technique to measure the cutting forces was developed for this study. The composite material developed in this study were embedded in acrylic (Kemdent Simplex Rapid, Associated Dental Products Ltd., UK) blocks encompassing a 3D printed mould for further use. Once set, the samples were mounted to a 3-axis load cell (Model 3A60A, Interface Force Measurements Ltd., UK). A high-speed dental handpiece (TE-95 BC Alegra Dental Air Rotor Handpiece, The W&H Group, Austria) with a cylindrical diamond bur (111-012 M, Dentsply Sirona, USA) was mounted to a vertical stage (LMS-180 Precision Linear Stage, Physik Instrumente GmBH, Germany), which in turn was mounted to a horizontal stage (Fig. 6). Both stages were controlled using an A-81 × PIglide Motion Controller (Physik Instrumente Ltd., Germany) running PIMikroMove (Version 2.10, 2015; Physik Instrumente GmBH, Germany). The handpiece was connected and powered by a portable turbine unit (GXJ Lab, China) and kept at a constant speed of 40,000 rpm. The rate at which the dental handpiece cut into the sample was kept at a set rate of 0.1 mm.s^-1^, with the forces being measured in real-time. Load data was recorded via a 4-channel signal amplifier (ME-Meβsysteme GmbH, Germany) connected to a computer running GSVmulti (Version 1.40, 2018; ME-Meβsysteme GmbH, Germany) which records real-time load acting on the 3D printed composites when the dental handpiece is moved into contact. The data is displayed as a force against time plot (Fig. 7).

Experiments were conducted to measure the force acting on the 3D printed composites as the dental handpiece was moved into contact using the positional stages. The dental handpiece was attached to the vertical stage that was subsequently mounted to the horizontal stages to allow cutting in the *X* direction (mesiodistal) and *Z* direction (occlusal). The handpiece was positioned 10 mm away from the sample before each cut to allow sufficient time for the bur to reach operational speed before contacting the samples’ surface. The handpiece cut in the mesiodistal direction as the horizontal stage traversed through the sample and 10 mm beyond the samples’ surface. The handpiece was then raised 10 mm upwards after the cut to ensure no contact was made with the sample. Completion of the cut through the tooth was followed by the horizontal stage returning to the original position before the cut (10 mm away from the sample) in the *X*-axis as, as well as the original position in the *Z* plane. The handpiece was translated downwards by 1 mm with respect to the previous cut and the cutting procedure of translating the bur along the *X*-axis to remove further tooth material was repeated. For all samples, an initial 1 mm deep cut was established with the dental handpiece (1 mm beginning from the highest point on the occlusal surface) to establish a flat surface for subsequent cuts to be compared. The first cut would record a force variation that reflected the geometry of the occlusal surface (Fig. 7, Cut 1) such that cutting through high features of the tooth resulted in high forces recorded whereas lower regions resulted in smaller recorded forces. The first cut was omitted from the data analysis to enable the comparison of material response to the cutting process and remove morphology influences. Following the initial cut, the force readout was seen to plateau (Cut 2 & 3) as the bur made contact with solid enamel. In some cases, which depended on the size of the tooth, the fourth cut caused the bur to contact both enamel and dentine, resulting in a fluctuation of cutting forces as the bur moved between the two materials. Subsequent cuts would then follow Cuts 2 & 3 in terms of presenting a force plateau as the bur contacted the solid dentine.

Force data were collected and later processed to establish an average force acting between the 3D printed composite and the dental handpiece by taking the numerical mean of all the force data points collected. Specifically, the points recorded between the 5th and 95th percentile were averaged to remove the points collected during the entrance and exit cuts, which were heavily affected by the external geometry of the tooth. The force collection process was additionally repeated on the extracted teeth so that a subsequent comparison could be made with the 3D printed typodont teeth.

### Data analysis

Data analysis was carried out using Microsoft Excel (Version 1909, 2019; Microsoft, USA) using a data analysis plugin. The data were subjected to one-way ANOVA test and, where relevant, a Tukey post hoc test to calculate the significance of the results, with statistical significance measured as *P* < 0.05.

## Data Availability

The datasets generated during and/or analysed during the current study are available from the corresponding author on reasonable request.

## References

[1] Loyaga-Rendon PG (2007). Compositional characteristics and hardness of acrylic and composite resin artificial teeth. J. Prosthet. Dent..

[2] Höhne, C., Rammler, T. & Schmitter, M. 3D printed teeth with included veneer preparation guide.* J. Prosthodont.* **30**(1), 51–56 (2021). 10.1111/jopr.13250 32869400

[3] Reymus M (2018). 3D Printed Replicas for Endodontic Education. Int. Endod. J..

[4] Lee B (2021). Dental students’ perceptions on a simulated practice using patient-based customised typodonts during the transition from preclinical to clinical education. Eur. J. Dent. Educ..

[5] Höhne C, Schwarzbauer R, Schmitter M (2019). 3D printed teeth with enamel and dentin layer for educating dental students in crown preparation. J. Dent. Educ..

[6] Kolling M (2021). Students’ perception of three-dimensionally printed teeth in endodontic training. Eur. J. Dent. Educ..

[7] Gutiérrez-Salazar MP, Reyes-Gasga J (2003). Microhardness and chemical composition of human tooth. Mater. Res..

[8] Bacali C (2019). The influence of graphene in improvement of physico-mechanical properties in PMMA denture base resins. Materials.

[9] Lee HH, Lee CJ, Asaoka K (2012). Correlation in the mechanical properties of acrylic denture base resins. Dent. Mater. J..

[10] Preis V (2018). Contact wear of artificial denture teeth. J. Prosthet. Res..

[11] Clements JL (2018). Do denture processing techniques affect the mechanical properties of denture teeth?. J. Prosthet. Dent..

[12] Cevik P, Yildirim-Bicer AZ (2016). The effect of silica and prepolymer nanoparticles on the mechanical properties of denture base acrylic resin. J. Prosthodont..

[13] Bera O (2011). Preparation and thermal properties of polystyrene/silica nanocomposites. Thermochim. Acta.

[14] Reymus M (2020). A critical evaluation of the material properties and clinical suitability of in-house printed and commercial tooth replicas for endodontic training. Int. Endod. J..

[15] Al-Sudani DI, Basudan SO (2016). Students' perceptions of pre-clinical endodontic training with artificial teeth compared to extracted human teeth. Eur. J. Dent. Educ..

[16] Tchorz JP (2015). Pre-clinical endodontic training with artificial instead of extracted human teeth?: does the type of exercise have an influence on clinical endodontic outcomes?. Int. Endod. J..

[17] dos Luz D (2015). Preparation time and perceptions of Brazilian specialists and dental students regarding simulated root canals for endodontic teaching: a preliminary study. J. Dent. Educ..

[18] Amini S, Miserez A (2013). Wear and abrasion resistance selection maps of biological materials. Acta Biomater..

[19] Senatov FS (2016). Mechanical properties and shape memory effect of 3D-printed PLA-based porous scaffolds. J. Mech. Behav. Biomed. Mater..

[20] Corcione CE (2017). The feasibility of printing polylactic acid–nanohydroxyapatite composites using a low-cost fused deposition modeling 3D printer. J. Appl. Polym. Sci..

[21] Mitsouras D (2015). Medical 3D printing for the radiologist. Radiographics.

[22] Rengier F (2010). 3D printing based on imaging data: review of medical applications. Int. J. Comput. Assist. Radiol..

[23] Parwani R (2017). Morphological and mechanical biomimetic bone structures. ACS Biomater. Sci. Eng..

[24] Cresswell-Boyes AJ (2018). Approaches to 3D printing teeth from X-Ray microtomography. J. Microsc..

[25] Miserez A (2007). Jumbo squid beaks: Inspiration for design of robust organic composites. Acta Biomater..

[26] Davis GR, Elliott JC (2003). High Definition X-ray microtomography using a conventional impact X-ray source. J. Phys. IV (Proceedings).

[27] Davis GR, Evershed ANZ, Mills D (2013). Quantitative high contrast X-ray microtomography for dental research. J. Dent..

[28] Feldkamp LA, Davis LC, Kress JW (1984). Practical cone-beam algorithm. J. Opt. Soc. Am. A-Opt. Image Sci. Vis..

[29] Landi E (2003). Carbonated hydroxyapatite as bone substitute. J. Eur. Ceram. Soc..

[30] Lefebvre L (2008). Sintering behaviour of 45S5 bioactive glass. Acta Biomater..

[31] Rashwan M, Cattell MJ, Hill RH (2019). The effect of barium content on the crystallization and microhardness of barium fluormica glass-ceramics. J. Eur. Ceram. Soc..

